# Molecular Alterations in Intraductal Carcinoma of the Prostate

**DOI:** 10.3390/cancers15235512

**Published:** 2023-11-22

**Authors:** Marit Bernhardt, Glen Kristiansen

**Affiliations:** Institute of Pathology, University Hospital Bonn, 53127 Bonn, Germany; marit.bernhardt@ukbonn.de

**Keywords:** intraductal carcinoma of the prostate, cribriform prostate cancer, intraductal proliferation

## Abstract

**Simple Summary:**

Intraductal carcinoma of the prostate (IDC-P) is a neoplastic proliferation within the prostatic ducts with a retained basal cell layer that exceeds the usual extent of precursor lesions. IDC-P may be present either in association with high-grade prostate cancer or independently of any high-grade cancer or invasive tumor. In this review, we focus on molecular alterations that are present in IDC-P with or without concomitant high-grade cancer as well as genetic alterations present in men that may lead to the development of IDC-P.

**Abstract:**

Intraductal carcinoma of the prostate is most commonly associated with high-grade invasive prostate cancer. However, isolated IDC-P without adjacent cancer or high-grade cancer is also well known. Common genetic alterations present in IDC-P with adjacent high-grade prostate cancer are those described in high-grade tumors, such as PTEN loss (69–84%). In addition, the rate of LOH involving *TP53* and *RB1* is significantly higher. IDC-P is common in the TCGA molecular subset of *SPOP* mutant cancers, and the presence of *SPOP* mutations are more likely in IDC-P bearing tumors. IDC-P without adjacent high-grade cancers are by far less common. They are less likely to have PTEN loss (47%) and rarely harbor an ERG fusion (7%). Molecular alterations that may predispose a person to the development of IDC-P include the loss of *BRCA2* and *PTEN* as well as mutations in *SPOP*. However, the causative nature of these genetic alterations is yet to be validated.

## 1. Introduction

In urological pathology, intraductal carcinoma of the prostate (IDC-P) is a notable topic of investigation. It is characterized by neoplastic proliferation within the prostatic ducts while retaining the basal cell layer, which, by definition, has to be lost in order to diagnose an invasive process. When compared to the prostate cancer precursor lesion referred to as high-grade prostatic intraepithelial neoplasia (HGPIN), which similarly presents a retained layer of basal cells, IDC-P exceeds the usual extent of HGPIN in regard to both size and atypia. The precise identification of IDC-P on core needle biopsies and resection specimens is of utmost importance, as its presence has consistently been linked to more aggressive tumor behavior, resulting in significantly worse prognoses and shorter overall survival times [1,2].

It is worth noting that although the concept of IDC-P was previously discussed and promoted in earlier medical literature, it only received formal recognition and classification as a distinct diagnostic entity in the 4th edition of the World Health Organization (WHO) classification in the year 2016 [3,4,5]. This acknowledgment by the WHO framework solidified IDC-P’s position as a crucial consideration in the field of prostate pathology. The current issue of the WHO classification (*Urinary and Male Genital Tumours*, WHO Classification of Tumours, 5th edition), provides a clear definition of IDC-P. Herein, IDC-P is defined as a complex proliferation, characterized by the (partial) preservation of the basal cell layer and the presence of a solid or densely cribriform architectural pattern. However, it is important to note that in cases where the architectural patterns are less complex, the presence of marked cytomorphological atypia, either with or without concurrent comedo-type necrosis, also justifies a diagnosis of IDC-P [2].

In comparison to the current, somewhat softer diagnostic criteria for the diagnosis of IDC-P, in the previous WHO classification (4th edition), the most widely accepted definition of IDC-P was considerably narrower in scope. In this definition, only lesions that fully met specific criteria were considered as part of the entity. The criteria comprised of ducts with dilation surpassing twice the normal size, growth patterns exhibiting solid, cribriform, or micropapillary characteristics, and a nuclear size expanding to six times the normal dimensions [3,6,7]. Lesions that did not precisely meet the criteria of IDC-P but exhibited a level of atypia, exceeding that observed in high-grade PIN (HGPIN), were categorized by many experts as “atypical lesions with suspicion of intraductal carcinoma” (ASID), “atypical intraductal proliferation” (AIP), or “intraductal cribriform proliferations”. Within the current WHO classification, AIP is the preferred term [8,9,10,11].

**Figure 1:**
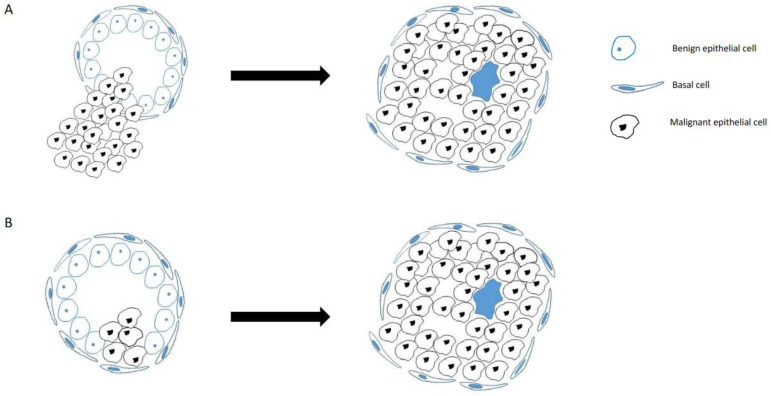
Schematic figure of concomitant IDC-P with invasion by a high-grade tumor (**A**) into pre-existing benign glands. In contrast, isolated IDC-P develops from a pre-existing benign duct (**B**) with dilation and retention of basal cell layer.

However, despite the substantial strides made in understanding the molecular characteristics, a fundamental aspect continues to be a matter of debate within the scientific community: the origin of IDC-P. Despite numerous investigations, the definite origin of IDC-P remains unclear. In its initial description, IDC-P was often conceived as an intraductal spread originating from an immediately adjacent invasive prostate cancer of a higher grade, a theory supported by early but also some current research [12,13,14,15]. Nonetheless, the emergence of rare cases, where isolated IDC-P is detected without any concurrent high-grade or invasive cancer, has inevitably raised profound questions about the previously accepted theories. This contradictory observation has led several experts in the field to propose a crucial distinction, one that, as mentioned, differentiates between concomitant (“conventional”) IDC-P and its isolated counterpart, often referred to as “precursor type” IDC-P, conceptualizing these as two distinct entities with unique clinical and pathological implications (Figure 1) [16].

In conclusion, intraductal carcinoma of the prostate (IDC-P) is a multifaceted area of study that is a matter of ongoing debate in urological pathology. In this review, we provide a short and concise overview of the aforementioned scenarios and their distinct molecular landscapes.

## 2. Molecular Alterations in IDC-P Cases with Concomitant Invasive Prostate Cancer

With regard to the landscapes of the molecular aberrations observed in cases of intraductal carcinoma of the prostate (IDC-P) concomitantly occurring with invasive prostate cancer, a complex interplay of genetic events is unveiled, exerting profound influence over tumor behavior, diagnostic intricacies, and potential therapeutic approaches.

IDC-P, a prominent entity, is encountered in approximately 20% of radical prostatectomy specimens, although it can be identified in up to 3% of prostate biopsies only [14,16]. Often, the contiguous prostate cancer manifests with a high-grade phenotype characterized by a Gleason pattern 4 or 5, thereby underscoring the clinical significance of IDC-P within the purview of prostate pathology. However, the diagnostic milieu is not devoid of challenges, particularly in the correct distinction of IDC-P and cribriform Gleason pattern 4. The crucial differentiation pertaining to the presence or absence of a basal cell layer, a pivotal diagnostic hallmark, is not invariably evident through conventional hematoxylin and eosin staining techniques [6]. The feasibility of a large-scale immunohistochemical analysis for routine clinical application remains constrained, leading to a more pragmatic approach wherein IDC-P and cribriform Gleason pattern 4, owing to their analogous prognostic implications, are frequently evaluated concomitantly. Furthermore, the selective choice of discrete loci for a genetic investigation mandates the labor-intensive and time-consuming procedure of microdissecting slides, thereby adding even more time and effort to the case [17]. Case series, in which tissue is microdissected to specifically obtain IDC-P for further molecular analysis, are typically small. However, the data obtained from DNA sequencing suggest a phylogenetic relationship between IDC-P and adjacent cancers in the majority of cases. Interestingly, in one case series, IDC-P and tumor metastasis shared the same ancestor, underlying the aggressiveness of the disease [15,18].

A significant recurrent genetic event in prostate cancer, *TMPRSS2::ERG* fusion, occurs in approximately 50% of all cases [19]. Interestingly, the rate of ERG fusion-positive cases in IDC-P slightly to strongly exceeds this range, ranging from 55% to 75% [8,20,21]. However, this does not appear to be the case in the Asian population. In a large cohort of >600 biopsies of treatment-naïve prostate cancers from Chinese patients, Nie et al. were only able to confirm ERG positivity in 16.7% of all prostate cancer cases. In contrast to the previous findings in the literature, the amount of ERG fusion-positive cancers among those harboring IDC-P was even lower, compromising only 10%. Interestingly, the proportion of IDC-P-positive tumors in the cohort was as high as 20% [22]. Given that the materials used for the study were core needle biopsies but not resection specimens, the discrepant result may be due to patient ethnicity but also the cohort itself. However, ethnic differences, which have not yet been subject or interest in IDC-P research do need to be further elucidated. Regarding the mechanisms driving ERG fusions, a suggestive observation from a previous study postulates that these may be different in IDC-P patients. Specifically, it has been suggested that IDC-P cases are more likely to gain ERG fusions through the deletion of interposed gene segments, a phenomenon associated with more aggressive tumors, as opposed to the more common mechanism of gene insertion [20].

*PTEN*, a tumor suppressor gene, is frequently lost in prostate cancers [23]. Notably, PTEN loss is recognized as a late event in tumorigenesis and is typically associated with late- and/or high-stage cancers. It is present in approximately 20–40% of all prostate cancers and an even higher 40% of metastatic tumors [24,25]. The loss of PTEN has significant implications, as it contributes to tumorigenesis through the deregulation of the oncogenic PI3K-AKT-mTOR pathway [25]. Beyond its role as a prognostic biomarker, the status of PTEN holds promise as a predictor of a treatment response to targeted therapies, positioning it as a potential predictive biomarker in the realm of prostate cancer [26,27]. In the context of IDC-P, PTEN loss is observed in more than 2/3 of cases, ranging from 69% to 84%, and reflecting a higher frequency compared to invasive prostate cancer [8,24,28]. Given the higher proportion of PTEN loss in IDC-P, PTEN is often utilized to emphasize the theory of IDC-P representing a later event in prostate cancer evolution. Additionally, given the rarity of PTEN loss in high-grade prostatic intraepithelial neoplasia (HGPIN), its utilization in differentiating between HGPIN and IDC-P has been the focus of extensive study [8,10,29]. At this point, it deserves to be mentioned that the authors have questioned the concept of HGPIN being exclusively a precursor lesion, as it may well represent an intraductal spread of an adjacent low-grade prostate cancer in many cases [30].

The concordance of both ERG and PTEN status between IDC-P and adjacent prostate cancer generally demonstrates good agreement, with reports indicating that this concordance may be present in up to 100% of cases. This observation lends empirical validation to the hypothesis of a clonal interconnection between IDC-P and an accompanying prostate cancer, particularly within the domain of “classical” (concomitant) IDC-P. This substantiates the proposition that these entities are closely linked, reinforcing their intimate genetic relationship [9,10,29].

In contrast to various other prevalent cancer types, prostate cancer is distinguished by a relatively diminished frequency of point mutations. However, this disparity in mutational burden is offset by a heightened incidence of genomic instability, which is characterized by an elevated prevalence of deletions, amplifications, and chromosomal rearrangements within the genome [31]. It is noteworthy that the extent of the loss of heterozygosity (LOH) in these patients with prostate cancer, concomitant with IDC-P, is demonstrably more pronounced than that observed in cases harboring solely pure acinar adenocarcinomas [32]. Upon scrutinizing the genetic milieu for genes influenced by LOH, IDC-P manifests a considerably augmented frequency of implication in *TP53* (60% in IDC-P in contrast to 40% in invasive prostate cancer) and *RB1* (81% compared to 60%) [33].

The analysis of The Cancer Genome Atlas (TCGA) cohort of prostate cancers has revealed a molecular classification that defines seven distinct molecular subgroups through cluster analysis. These subgroups are defined based on the presence of specific genomic events, including fusions, mutations, or other alterations. Notably, a substantial proportion of prostate cancers, up to 74%, can be attributed to fusions found in ETS-family genes (*ERG*, *ETV1*, *ETV4*, and *FLI1*) or mutations in genes such as *SPOP*, *FOXA1*, or *IDH1* [34]. A noteworthy finding within this molecular classification is the significantly higher prevalence of IDC-P in prostate cancers within the molecular class characterized by *SPOP* mutant cancers, accounting for up to 90% of cases within this group. However, the distribution of IDC-P among the other molecular classes does not appear to differ significantly from that of “conventional” prostate cancer, suggesting that while the *SPOP* mutation is strongly associated with IDC-P cases, additional factors contribute to the observed molecular heterogeneity [35].

When considering mutations in IDC-P beyond the aforementioned *SPOP* mutation, which is present in 17% to 29% of IDC-P cases but found in only 10% to 11% of all prostate cancer cases, IDC-P shows a higher prevalence of mutations in genes associated with prostate cancer aggressiveness. These genes include *ATM* (7.3% in IDC-P vs. 1% in all prostate cancers), *FOXA1* (15% vs. 10%), and *TP53* (19% vs. 10%) (Table 1) [34,35,36].

In addition to changes on a DNA level, the molecular landscape of IDC-P also involves RNA alterations. Here, long non-coding RNAs (lncRNAs) are of particular interest. LncRNAs are RNAs of at least 200 bp in length that do not code for a specific protein. These molecules have been shown to play significant roles in cell processes, including gene expression and cell cycle regulation. Moreover, certain lncRNAs have been implicated in increasing proliferation and metastatic capacity in tumors. In the context of prostate cancer, the upregulation of specific lncRNAs, such as *UAC1*, *PVT1*, *PCA3*, *HOTAIR*, and *SChLAP1*, has been associated with increased tumor cell growth [37]. *SChLAP1* (second chromosome locus associated with prostate-1) is particularly interesting—lncRNA expressed in the nucleus. A high expression of *SChLAP1* can be found in approximately 25% of all prostate cancers except for 44% of prostate cancers harboring IDC-P or Gleason pattern 4 of the cribriform type. Its presence has been linked to adverse prognostic features, such as metastasis and a higher Gleason grade. In addition, it serves as an independent risk factor for the prediction of biochemical recurrence. Furthermore, *SChLAP1* expression is significantly higher in tumors harboring IDC-P or cribriformous Gleason pattern 4 when compared to lower Gleason grades or different morphologies of Gleason pattern 4 [38,39,40,41,42].

*Epigenetics* refers to changes in gene function that can be passed down through mitosis and meiosis but are not a result of alterations in the DNA sequence. One of the mechanisms playing an important role in epigenetic modification is the methylation of DNA, which frequently occurs in CpG dinucleotide-rich regions [43]. The methylation of DNA in these cases often leads to decreased transcription of the corresponding gene segment. Multiple CpGs, so-called CpG islands, are commonly located in the vicinity of promoters, transcription start sites, or non-coding gene segments. Alterations in the tumor methylome typically result in enhanced methylation at distinct promoters and a decreased overall methylation. Thus, tumors develop a distinct pattern of hyper- and hypomethylated gene segments, which can usually be assigned to defined entities or lead to the definition of new entities or subtypes of existing entities [44]. Methylation analysis indicated that many of the genes with alterations in methylation in both invasive prostate cancer and IDC-P are part of androgen receptor transcriptional regulation, androgen response, and genes that are targets of Myc. Furthermore, approximately 20,000 genes display varying methylation patterns when comparing IDC-P and invasive prostate cancer. These genes belong to the androgen receptor pathway. In addition, TNFα, TGFβ, and Notch signaling have also been observed to be susceptible to methylation in IDC-P. Based on the data obtained from the integration of methylation and transcriptome analysis, Zhao et al. developed a transcriptional signature consisting of only eight genes (*SCD*, *SQLE*, *GMNN*, *TPH2*, *TBC1D31*, *NIPAL1*, *YES1*, and *FANCF*) to distinguish prostate cancer cases with IDC-P from those without [15].

## 3. Molecular Alterations in Isolated IDC-P

The presence of intraductal carcinoma of the prostate (IDC-P) unaccompanied by concomitant prostate cancer on a core needle biopsy represents an exceedingly uncommon scenario, manifesting within a marginal fraction of instances. To be precise, the manifestation of isolated IDC-P constitutes a meager proportion of less than 0.5% within the entire spectrum of biopsy cases, thereby underscoring its distinct rarity [16,21]. However, an interesting observation arises upon closer examination of subsequent radical prostatectomy specimens. Surprisingly, a substantial subset of these specimens, reaching an incidence as notable as 10%, exclusively manifests the presence of IDC-P [45]. Although such occurrences are infrequent, they harbor a unique feature due to the definite assurance that the discernible IDC-P does not originate from a retrograde dissemination of a preexisting high-grade prostate cancer. The criteria for inclusion also encompass scenarios with invasive prostate cancer present in the organ. In these, the infiltrative malignancy assumes a low-grade phenotype, delineated by a maximal Gleason pattern of 3 + 3 = 6. This precondition acknowledges the implausibility of the retrograde spread of a high-grade malignancy within pre-established ductal structures [46]. In order to differentiate this particular manifestation of IDC-P from the conventional iteration characterized by an adjacent invasive carcinoma, it is judiciously denoted as “isolated IDC-P” or “precursor type IDC-P” [47]. Significantly, the isolated IDC-P variety is distinctive in its prognostic value, demonstrating an extended interval of progression-free survival when compared with the conventional IDC-P featuring neighboring invasive carcinoma [48].

Investigating the clonal relationship between tumor foci in prostate cancer often involves immunohistochemical analyses of ERG and PTEN proteins. As mentioned above, in the context of conventional IDC-P, these markers demonstrate a high concordance between the foci of IDC-P and invasive cancer [48]. However, this scenario shifts when considering isolated IDC-P in conjunction with concomitant low-grade cancer. In these cases, the reported concordance is notably lower, with 67% for ERG and 56% for PTEN. This discrepancy raises the assumption that isolated IDC-P may indeed represent an independent neoplasm, displaying a distinct genetic and molecular profile [38]. It is worth noting that the detection rate of ERG in isolated IDC-P is a mere 7%, which is significantly lower than in “normal” prostate cancer. However, this contrasts the more prevalent PTEN loss, which (in isolated IDC-P) is within the range observed in invasive prostate cancer without IDC-P, albeit significantly lower than in conventional IDC-P [19,46].

In contrast to the more commonly observed alterations of ERG and PTEN, which do seem to differ in frequency between concomitant and isolated IDC-P, mutations as defined by the aforementioned TCGA subgroups tend to be present in similar proportions. Mutation frequencies have been reported in 14% of isolated IDC-P cases for *SPOP* and 29% for *FOXA1* [46]. However, these data should be treated with caution, as the already limited amount of data available for concomitant IDC-P are even more limited for the isolated form.

More recent work comparing the clonal relationship between benign prostate tissue, invasive prostate cancer, and IDC-P with whole exome sequencing has been able to support the theory of the different types of IDC-P. Invasive cancer and concomitant IDC-P were shown to be clonally distinct from each other in up to 22% of cases, meaning that they originated from independent precursors. It should be noted, however, that tumors labeled as clonally distinct with molecular analysis did not always, although mostly, meet the histomorphological criteria of isolated IDC-P [15].

## 4. Molecular Alterations Present in Patients Prone to Develop IDC-P

The primary focus of research on intraductal carcinoma of the prostate (IDC-P) has historically revolved around somatic alterations within IDC-P itself or its association with prostate cancer in the context of IDC-P. Nonetheless, a subset of studies has explored the genetic changes that may underlie the emergence of IDC-P, shedding light on potential genetic factors contributing to its development. Given that genetic risk factors can be attributed to a substantial portion of prostate cancers, namely up to 57%, it is reasonable to consider the presence of such factors in IDC-P as well [49].

Hereditary prostate cancers are characterized by mutations in genes such as *HOXB13*, *BRCA2*, and *ATM*. However, it has to be mentioned that *BRCA* and *ATM* mutations can occur even in cases without a family history, accounting for up to 60% of such instances [2]. Both *BRCA2* and *ATM*, amongst others, are involved in DNA damage repair through a homologous recombination (HRD). Germline HRD defects are found in up to 12% of metastasized prostate cancer cases and up to 4.5% of localized tumors [50]. Hereof, mutations in *BRCA2* stand as the most prevalent germline alteration in prostate cancer and increase the risk of cancer development by as much as five-fold [49,51,52]. Both somatic and germline *BRCA2* mutations can be detected in up to 10% of all prostate cancers. Specifically, the germline mutation is present in 4.5% of all cases and rises up to 5.3% in metastatic diseases. Importantly, the presence of a *BRCA2* germline mutation is independently associated with more aggressive cancers and a poorer prognosis [49,51,53].

Notably, significantly higher proportions of IDC-P have been demonstrated within in vitro models using xenografts of *BRCA2* germline carriers, suggesting a plausible role for *BRCA2* as an inducer of intraductal growth [54]. The association between *BRCA2* and IDC-P observed in vitro has also been validated in vivo in patient cohorts [55]. As a result, the recognition of IDC-P in prostate cancer patients has been incorporated as a rationale for performing germline genetic testing in the United States, although this practice is not yet part of the European guidelines [17,51,52]. However, more recent publications have not consistently confirmed the previously mentioned association between germline *BRCA2* mutations and the presence of IDC-P upon the development of prostate cancer. In contrast, Lozano et al. found IDC-P to be less prevalent in a cohort of germline *BRCA2* carriers with a proportion of 36% IDC-P-positive cases in the carrier, versus 50% in the non-carrier group. Still, bi-allelic loss of *BRCA2* was found to significantly correlate with IDC-P, suggesting the need for further research to gain a better understanding of the underlying mechanisms [53].

In animal models utilizing mutant mice, a cribriform pattern of carcinoma has been described in *SPOP* mutant transgenic mice in the presence of a monoallelic loss of *PTEN* (*PTEN^L/+^*), while this pattern is notably absent in *PTEN* wild-type (*PTEN^wt^*) mice. Furthermore, mice with a bi-allelic loss of *PTEN* (*PTEN^L/L^*) were more prone to developing prostate cancer with a cribriform morphology. Given these findings, in combination with the increased prevalence of IDC-P in the molecular subgroup of *SPOP* mutant cancers and the higher proportion of *PTEN* loss observed in IDC-P, these genetic events may also play a significant role as contributing factors in the development of IDC-P [35].

## 5. Summary

Indeed, the molecular landscape of intraductal carcinoma of the prostate (IDC-P) often reveals genetic alterations that are commonly associated with high-grade cancers, such as the loss of PTEN or mutations in *TP53*. Additionally, there is a significant elevation in the frequency of *BRCA2* mutations in patients with IDC-P. However, a notable distinction emerges when considering cases of “isolated IDC-P,” where IDC-P is present without adjacent high-grade cancer. In this scenario, there is a notable reduction in the molecular changes typically associated with high-grade cancer. This contrast suggests that isolated IDC-P may represent a distinct and independent entity within the spectrum of prostate cancer. Molecular changes usually associated with high-grade prostate cancer, such as *PTEN* loss or *TP53* mutations, are enriched in IDC-P which may explain its aggressive clinical characteristics. Additionally, the possibly heightened rate of *BRCA2* mutations in IDC-P patients further adds complexity to the genetic profile of this subtype of prostate cancer. These molecular findings reinforce the need for a thorough molecular characterization to guide clinical decisions and treatment strategies for patients with IDC-P, particularly those with concurrent high-grade features. On the other hand, the notable absence or the reduced frequency of these molecular changes in isolated IDC-P, the variant of IDC-P without adjacent high-grade cancer, raises intriguing questions about its distinct nature. The reduced presence of high-grade cancer-associated genetic alterations in isolated IDC-P suggests that it might have a distinct origin or biological behavior compared to conventional IDC-P, which occurs alongside high-grade cancer. This difference in the molecular profile between isolated IDC-P and its counterpart implies that these two subtypes might represent separate entities along the spectrum of prostate cancer progression.

## 6. Future Directions

Further research is essential to unravel the mechanisms underlying the development and progression of IDC-P, particularly in the context of isolated IDC-P. Investigating the genetic and molecular differences between these subtypes will not only enhance our understanding of IDC-P, but may also lead to more precise diagnostic and therapeutic approaches tailored to the unique characteristics of each subtype. The distinct molecular profile of isolated IDC-P highlights its potential as a promising avenue for targeted research, ultimately leading to more effective treatment and improved outcomes for patients with this specific form of prostate cancer. Given that the number of review articles appears to exceed the number of original research articles, it is clear how much is still unknown and how important additional data would be.

## Figures and Tables

**Table 1:** Overview on common aberrations and their frequency in both isolated and concomitant IDC-P.

Alteration	Isolated IDC-P	%	Concomitant IDC-P	%
Fusion	TMPRSS2::ERG	7	TMPRSS2::ERG	55–75
Mutation	SPOP	14	SPOP	17–29
	FOXA1	29	FOXA1	15
			TP53	19
			ATM	7
Loss	PTEN	47	PTEN	69–84
LOH			TP53	60
			RB1	81
lncRNA upregulation			SChLAP1	44

## Data Availability

The data can be shared up on request.
